# Spatiotemporal features for asynchronous event-based data

**DOI:** 10.3389/fnins.2015.00046

**Published:** 2015-02-24

**Authors:** Xavier Lagorce, Sio-Hoi Ieng, Xavier Clady, Michael Pfeiffer, Ryad B. Benosman

**Affiliations:** ^1^Equipe de Vision et Calcul Naturel, UMR S968 Institut National de la Santé et de la Recherche Médicale, Centre National de la Recherche Scientifique UMR 7210, Centre Hospitalier National d' Ophtalmologie des Quinze-Vingts, Université Pierre et Marie CurieParis, France; ^2^Institute of Neuroinformatics, University of Zürich and Eidgenössische Technische Hochschule (ETH) ZürichZürich, Switzerland

**Keywords:** echo-state networks, spatiotemporal, feature extraction, recognition, silicon retinas

## Abstract

Bio-inspired asynchronous event-based vision sensors are currently introducing a paradigm shift in visual information processing. These new sensors rely on a stimulus-driven principle of light acquisition similar to biological retinas. They are event-driven and fully asynchronous, thereby reducing redundancy and encoding exact times of input signal changes, leading to a very precise temporal resolution. Approaches for higher-level computer vision often rely on the reliable detection of features in visual frames, but similar definitions of features for the novel dynamic and event-based visual input representation of silicon retinas have so far been lacking. This article addresses the problem of learning and recognizing features for event-based vision sensors, which capture properties of truly spatiotemporal volumes of sparse visual event information. A novel computational architecture for learning and encoding spatiotemporal features is introduced based on a set of predictive recurrent reservoir networks, competing via winner-take-all selection. Features are learned in an unsupervised manner from real-world input recorded with event-based vision sensors. It is shown that the networks in the architecture learn distinct and task-specific dynamic visual features, and can predict their trajectories over time.

## 1. Introduction

Humans learn efficient strategies for visual perception tasks by adapting to their environment through interaction, and recognizing salient features. In contrast, most current computer vision systems have no such learning capabilities. Despite the accumulated evidence of visual feature learning in humans, little is known about the mechanisms of visual learning (Wallis and Bülthoff, 1999). A fundamental question in the study of visual processing is the problem of feature selection: which features of a scene are extracted and represented by the visual cortex? Classical studies of feature selectivity of cortical neurons have linked neural responses to properties of local patches within still images (Hubel and Wiesel, 1962; Olshausen and Field, 1997). Conventional artificial vision systems rely on sampled acquisition that acquires static snapshots of the scene at fixed time intervals. This regular sampling of visual information imposes an artificial timing for events detected in a natural scene. One of the main drawbacks of representing a natural visual scene through a collection of snapshot images is the complete lack of dynamics and the high amount of redundancy in the acquired data. Every pixel is sampled continuously, even if its output value remains unchanged. The output of a pixel is then unnecessarily digitized, transmitted, stored, and processed, even if it does not provide any new information that was not available in preceding frames. This highly inefficient use of resources introduces severe limitations in computer vision applications, since the largely redundant acquired information lead to a waste of energy for acquisition, compression, decompression and processing (Lichtsteiner et al., 2008).

Biological observations confirm that still images are largely unknown to the visual system. Instead, biological sensory systems are massively parallel and data-driven (Gollisch and Meister, 2008). Biological retinas encode visual data asynchronously through sparse firing spike trains, rather than as frames of pixel values (Roska and Werblin, 2003). Current studies show that the visual system effortlessly combines the various features of visual stimuli to form coherent perceptual categories relying on a surprisingly high temporal resolution: the temporal offsets of on-bistratified retina cells responses show an average standard deviation of 3.5 ms (Berry et al., 1997; Uzzell and Chichilnisky, 2004). Neurons in the visual cortex also precisely follow the temporal dynamics of the stimuli up to a precision of 10 ms. In order to bridge the gap between artificial machine vision and biological visual perception, computational vision has taken inspiration from fundamental studies of visual mechanisms in animals (Hubel and Wiesel, 1962; Wallis and Rolls, 1997). One main focus of these approaches have been various computational models of simple and complex cells in the primary visual cortex (V1) Hubel and Wiesel (1962); Fukushima (1980); Riesenhuber and Poggio (1999), which are characterized by their preferred response to localized oriented bars. Typically, this orientation-tuned response of V1 cells has been modeled with Gabor Filters (Gabor, 1946), which have been used as the first layer of feature extraction for visual recognition tasks (Huang et al., 2004; Ilonen et al., 2007). The most well-known example of biologically inspired, although still frame-based model of object recognition is the HMAX model (Riesenhuber and Poggio, 1999; Serre et al., 2006; Mutch et al., 2010). It implements a feedforward neural network based on a first layer of Gabor filters followed by different layers realizing linear and non-linear operations modeled on primate cortex cells. However, HMAX like other approaches implementing neural networks to perform visual tasks (Lin and Huang, 2005) are still based on processing still images and therefore cannot capture key visual information mediated by time.

This paper introduces an unsupervised system that allows to extract visual spatiotemporal features from natural scenes. It does not rely on still images, but on the precise timing of spikes acquired by an asynchronous spike-based silicon retina (Lichtsteiner et al., 2008). The development of asynchronous event-based retinas has been initiated by the work of Mahowald and Mead (Mead and Mahowald, 1988). Neuromorphic asynchronous event-based retinas allow new insights into the capabilities of perceptual models to use time as a source of information. Currently available event-based vision sensors (Delbruck et al., 2010; Posch et al., 2011) produce compressed digital data in the form of time-stamped, localized events, thereby reducing latency and increasing temporal dynamic range compared to conventional imagers. Because pixel operation is now asynchronous and pixel circuits can be designed to have extremely high temporal resolution, silicon retinas accomplish both the reduction of over-sampling of highly redundant static information, as well as eliminating under-sampling of very fast scene dynamics, which in conventional cameras is caused by a fixed frame rate. Pixel acquisition and readout times of milliseconds to microseconds are achieved, resulting in temporal resolutions equivalent to conventional sensors running at tens to hundreds of thousands of frames per second, without the data overhead of conventional high-speed imaging. The implications of this approach for machine vision can hardly be overstated. Now, for the first time, the strict temporal resolution vs. data rate tradeoff that limits all frame-based vision acquisition can be overcome. Visual data acquisition simultaneously becomes fast and efficient. A recent review of these sensors can be found in Delbruck et al. (2010) and Posch et al. (2014).

Despite the efficiency of the sensor representation, it is far from straightforward to port methods that have proven successful in computer vision to the event-based vision domain. Much of the recent success of computer vision comes from the definition of robust and invariant feature or interest point extractors and descriptors (Lowe, 1999, 2004; Bay et al., 2008). Although such methods have proven to be very useful for static image classification, they require processing of the whole image, and do not take temporal information into account. Dynamical features for event data should instead recognize features only from novel visual input, and recognize them as they appear in the sparse input stream. This requires a model that can continuously process spiking inputs, and maintain a representation of the feature dynamics over time, even in the absence of input. Here we present an architecture for feature learning and extraction based on reservoir computing with recurrent neural networks (Schrauwen et al., 2007), which integrate event input from neuromorphic sensors, and compete via a Winner-Take-All (WTA) technique to specialize on distinct features by predicting their temporal evolution.

A proof of concept for the performance of the architecture is demonstrated in three experiments using natural recordings with event-based vision sensors. In the first experiment, we present a set of oriented bars to the camera in order to the show the capacity of the model to extract simple features in an unsupervised manner, using a big spatial receptive field to emphasize the graphical visualization of the learnt features. In the second experiment, the full capacity of the method is demonstrated by mapping the field of view to several small receptive fields, and showing that the model is still capable of reliably extracting features from the scene. The last experiment applies the architecture to complex object features. All experiments were conducted with real-world recordings from DVS cameras (Lichtsteiner et al., 2008), and thus are subject to the standard noise distribution of such sensors.

## 2. Materials and methods

### 2.1. Event-based asynchronous sensors

**Figure 1:**
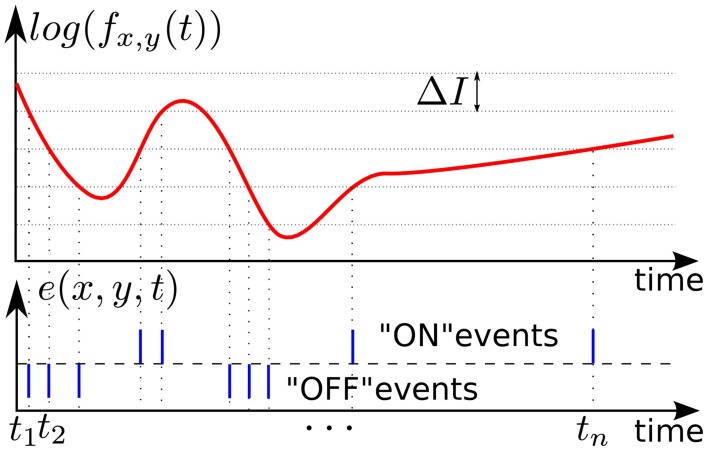
**Illustration of event-based encoding of visual signals**. Shown are the log-luminance measured by a pixel located at (*x*, *y*)^*T*^ and the asynchronous temporal contrast events signal generated by the DVS with respect to the predefined threshold Δ*I*.

In our experiments we used asynchronous event-based input signals from a Dynamic Vision Sensor (DVS) (Lichtsteiner et al., 2008), which mimics the biological retina in silicon. It encodes visual information using the Address-Event Representation (AER), and has a spatial resolution of 128 × 128 pixels. The DVS outputs an asynchronous stream of events that signal local relative luminance changes in the scene, at the time they occur. Each pixel works independently for its receptive field, and creates events whenever the local luminance change since the time of the last emitted event exceeds a given threshold Δ*I* on a logarithmic scale. The typical threshold is around 15% of relative contrast variation. If the change is an increase /decrease then an ON/OFF event is generated by the pixel (see Figure 1). This asynchronous way of coding allows to convey the timing of the events with a high temporal resolution (~1 μs). The “effective frame rate” of such pixels is several kHz. We define an event occurring at time *t* at the pixel (*x*, *y*)^*T*^ as:

(1) e(x, y, t)= |p| =1,

where *p* is the polarity of the event. *p* equals 1 (“ON”) whenever the event signals an intensity increase, or −1 (“OFF”) for a decrease, but for the purposes of this article the polarity is not used. This data-driven representation reduces redundancy in the visual input, and maintains the encoding of exact times of input signal changes, which allows very high temporal dynamics of acquisition.

### 2.2. General architecture

**Figure 2:**
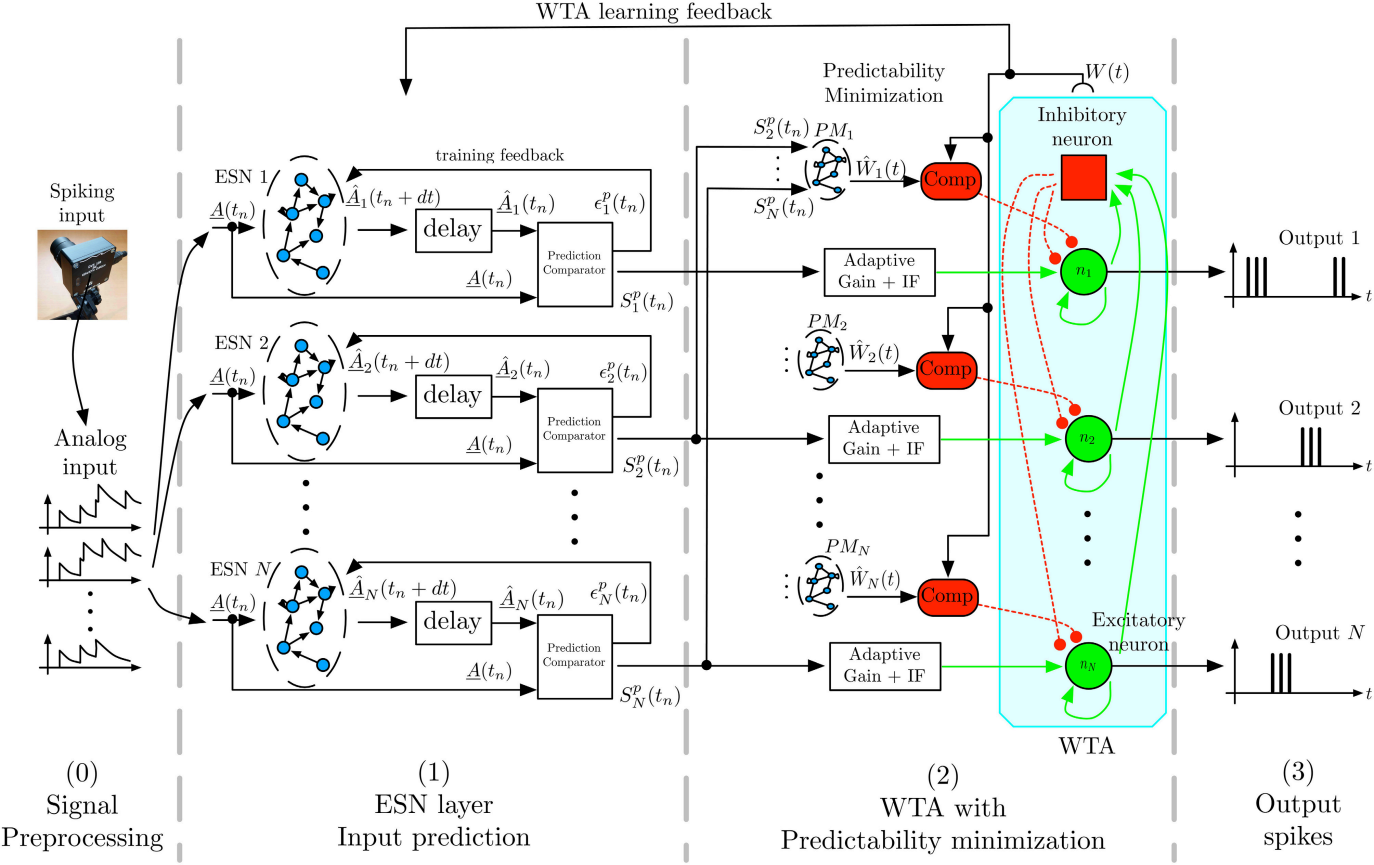
**Architecture for unsupervised spatiotemporal feature extraction**. Spikes from the DVS are transformed by filtering into analog input signals that are sent to a set of ESN networks. Each ESN is trained to predict future input activations based on current and past activities. The prediction is compared to the actual inputs, and the output signal *S*^*p*^_*k*_, which is a representation of the ESN's prediction performance is fed into a Winner-Take-All (WTA) network. This WTA selects the best predicting ESN and enables it to train on the present input sequence. A predictability minimization process promotes orthogonality of predictions between the different ESNs during the WTA selection. The combination of temporal prediction and competition through the WTA allows each ESN to specialize on the prediction of a distinct dynamical feature, which thus leads to learning of a set of different feature detectors.

Figure 2 shows the general architecture of the feature selection process. In the following we briefly describe the overall architecture, with more detailed descriptions of the individual components below. To capture the temporal dynamics of spatiotemporal features, we use Echo-State Networks (ESN) (Jaeger, 2002) that act as predictors of future outputs. To achieve unsupervised learning of distinct features we use multiple ESNs that compete for learning and detection via a WTA network. As the first stage, the signal coming from the DVS retina is preprocessed, by converting the DVS output into analog signals as required by the ESNs' structure. In the second stage, labeled *ESN layer* in Figure 2, each ESN receives the converted output of the DVS to predict its evolution one timesteamp in the future. The readout of each ESN is trained for this task, and each network should learn to predict different temporal dynamics. To achieve this, the next layer of the architecture, labeled *WTA with Predictability minimization* in Figure 2, implements a Winner-Take-All (WTA) neural network, which selects the best predictor from the available set of predicting ESNs. Through competition, the WTA inhibits poorly predicting ESNs to ensure that the best predictor has sufficient time to learn a particular spatiotemporal sequence. This layer also contains a predictability minimization process to promote orthogonality of predictions between the different ESNs. The selected ESN is then trained to recognize the spatiotemporal pattern, and learns to predict its temporal evolution. The WTA competition ensures that each ESN specializes on an independent feature, thus preventing two ESNs from predicting the same pattern. Consequently, at any given time, the winning network in the WTA layer indicates the detected feature. Through random initialization of ESNs and WTA competition, the architecture extracts distinct spatiotemporal features from event-based input signals in a completely unsupervised manner.

For the experiments described in this article, the architecture has been fully implemented in software, using DVS recordings of real-world stimuli as inputs. In particular, the visual inputs for all experiments contain the typical noise for this kind of sensor, and do not use idealized simulated data.

### 2.3. Signal pre-processing

The DVS retina has approximately 16*K* pixels in total. Directly using each pixel as an input to the ESN reservoir would require a network with 16*K* input neurons, and, in typical reservoir computing setups, 10–100 times more hidden neurons. Since this is a prohibitively large size for real-time simulation of neural networks on conventional current computers, a pre-processing stage is introduced to downsample the dimensionality of the input. Please note that this is not a fundamental requirement, since especially future large-scale neuromorphic processors and other dedicated hardware platform could potentially handle real-time execution of such large networks (see Discussion), but this is beyond the scope of our proof-of-principle study.

**Figure 3:**
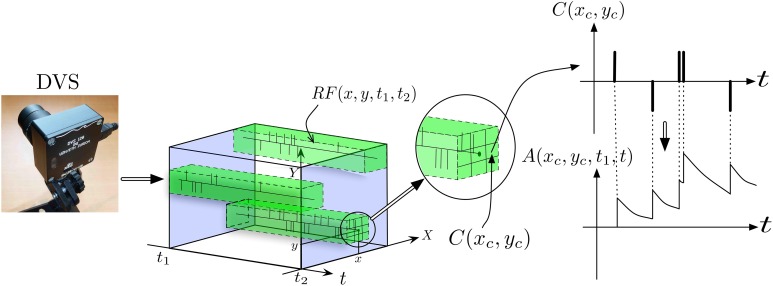
**Illustration of signal pre-processing to convert DVS events into equivalent analog input for ESNs**. In order to reduce the number of input channels to the system and reduce computational load, the input from retina pixels is first spatially subsampled into cells *C*(*x*_*c*_, *y*_*c*_). Each set of ESNs then receives input from a particular receptive field *RF*(*x*, *y*, *t*_1_, *t*_2_). To compute the equivalent analog input, an exponential kernel finally is applied to each event contained in the receptive field.

Figure 3 provides a more detailed view of the first layer of the architecture, named layer (0) in Figure 2. To reduce the input dimensionality of the DVS signal, the retina pixels are first spatially resampled into cells *C*(*x*_*c*_, *y*_*c*_) of δ_*x*_ × δ_*y*_ pixels, each integrating pixels around the center (*x*_*c*_, *y*_*c*_) according to:

(2) $$C(x_{c},y_{c})=\left\{(x,y)\;|\;\begin{array}{c} x\in [x_{c}-\delta_{x},x_{c}+\delta_{x}]\\ y\in [y_{c}-\delta_{y},y_{c}+\delta_{y}] \end{array}\right\}.$$

Next, the signals are quantized by introducing spatiotemporal receptive fields *RF*(*x*_0_, *y*_0_, *t*_1_, *t*_2_), covering Δ_*x*_ × Δ_*y*_ subsampling cells, which collect all events in a spatiotemporal volume in the time interval [*t*_1_, *t*_2_] according to:

(3) $$\begin{array}{l} RF(x_{0},y_{0},t_{1},t_{2})=\\ \left\{e(x,y,t)|t\in [t_{1},t_{2}],(x,y)\in C(x_{c},y_{c}),\;\begin{array}{l} |x_{c}-x_{0}|\leq \Delta_{x}\\ |y_{c}-y_{0}|\leq \Delta_{y} \end{array}\right\}. \end{array}$$

Conversion of events into analog signals is achieved by filtering with a causal exponential filter with time constant τ, defined as *G*(*t*, *t*_*i*_) = *e*^−(*t* − *t*_*i*_)/τ^ · *H*(*t* − *t*_*i*_), where *H*(*t*) is the Heaviside function, which is 1 for *t* ≥ 0 and zero otherwise. This filter is applied to all spikes coming from pixels (*x*, *y*) contained in a receptive field *RF*(*x*_0_, *y*_0_, *t*_0_, *t*), yielding the analog output signal *A*, which is fed into the ESNs:

(4) $$A(x,y,t_{0},t)=\sum_{\begin{array}{c} e(x_{i},y_{i},t_{i})\;\in \;RF_{\left(x_{0},y_{0},t_{0},t\right)}\\ x_{i}=x,\;y_{i}=y \end{array}}G(t,t_{i})\;\;\;,$$

where *t*_0_ is a chosen time origin.

The complete preprocessed input at time *t* fed into the ESN layer is the vector formed by all outputs *A*(*x*, *y*, *t*_0_, *t*) of pixels contained in *RF*(*x*_0_, *y*_0_, *t*_0_, *t*). For clarity, we will in the following only consider a single receptive field denoted as *A*(*t*):

(5) $$\underline{A}(t)=\left(\begin{array}{c} A(x_{1},y_{1},t_{0},t)\\ \vdots \\ A(x_{M},y_{M},t_{0},t) \end{array}\right)$$

### 2.4. ESN layer—input prediction

This layer (Figure 2-(1)) computes the prediction of input signals for *N* different ESNs (Jaeger, 2002). The *k*^th^ ESN is defined by its internal state *s*^*k*^, and the three weight matrices *W*^*k*^_out_ (for output or *readout* weights), *W*^*k*^_in_ (for input weights), *W*^*k*^_back_ (for feedback weights), and the recurrent weights *W*^*k*^_r_. These weight matrices are initialized randomly for each ESN and encoded as 64 bit floating-point numbers. The internal state *s*^*k*^ of the ESN and its output (*out*^*k*^) are iteratively updated, and evolve according to:

(6) $$\begin{array}{l} s^{k}(t_{n})=f(W_{\text{r}}^{k}\;\cdot \;s^{k}(t_{n\;-\;1})+W_{\text{in}}^{k}\cdot \underline{A}(t_{n})\\ \;+W_{\text{back}}^{k}\;\cdot \;out^{k}(t_{n\;-\;1})), \end{array}$$

(7) $$out^{k}(t_{n})\;=f^{\text{out}}\left(W_{\text{out}}^{k}\;\cdot \;s^{k}(t_{n})\right).$$

In our experiments, the logistic function is used as the non-linearity *f* for the internal state evolution, and a linear readout is used as *f*^out^. Every ESN is trained to predict its future input at one timestep ahead (i.e., at *t*_*n*_ + *dt*), thus the output of the ESN according to Equation 7 creates a prediction *Â*_*k*_(*t*_*n*_ + *dt*) = *out*^*k*^(*t*_*n*_), which should match *A*(*t*_*n*_ + *dt*). As is usual for ESNs, only the readout weights *W*^*k*^_out_ are adapted, the recurrent and other weights are kept at their random initial values which are drawn from uniform distributions.

As suggested in Jaeger and Haas (2004), training of the readout weights *W*^*k*^_out_ can be achieved with a standard recursive least squares algorithm (here a version described in Farhang-Boroujeny (2013) was used). This algorithm recursively adapts *W*^*k*^_out_ so as to minimize a weighted linear least squares cost function, computed from the prediction error:

(8) $$\epsilon_{k}^{p}(t_{n})=|{\underline{\hat{A}}}_{k}(t_{n})-\underline{A}(t_{n})|.$$

This method is well-suited for online learning, since the coefficients of *W*^*k*^_out_ can be updated as soon as new data arrives.

The output of the ESN layer into the subsequent WTA layer is a similarity measure *S*^*p*^_*k*_(*t*_*n*_) for each ESN, which indicates the quality of each prediction for the currently observed input:

(9) $$S_{k}^{p}(t_{n})=\frac{\sum_{i}\left|\underline{A}{\left(t_{n}\right)}_{i}.{\underline{\hat{A}}}_{k}{\left(t_{n}\right)}_{i}\right|}{\sum_{i}\left|\underline{A}{\left(t_{n}\right)}_{i}\right|\;.\;\sum_{i}\left|\underline{\hat{A}}{\left(t_{n}\right)}_{i}\right|},$$

where *i* is summing over all components of *A*(*t*_*n*_) and *Â*(*t*_*n*_), which have been properly normalized to take on values between 0 and 1.

### 2.5. Winner-take-all selection

Based on the indicators of prediction quality *S*^*p*^_*k*_(*t*_*n*_) computed by the ESN layer, the third layer of the model (Figure 2-(2)) selects the best predictor among the *N* ESNs through a WTA mechanism. The WTA network consists of a set of *N* neurons {*n*_1_, …, *n*_*N*_} plus an inhibitory neuron, which is recurrently and bi-directionally connected with the excitatory neurons, as detailed in Coultrip et al. (1992), Douglas et al. (1994), Liu and Oster (2006), and Oster et al. (2009). The task of the WTA is to select from the pool of ESNs the one whose prediction best matches the actual dynamics of the present input, and which thus has the highest similarity *S*^*p*^_*k*_(*t*_*n*_), as computed by layer (1) in Figure 2.

Inputs to the WTA neurons are generated from the *S*^*p*^_*k*_ values using non-leaky Integrate-and-Fire (IF) neurons, which transform the analog values into spike trains. To make the WTA network more robust to the variations in the similarity measure, a sigmoid function is applied to the *S*^*p*^_*k*_ values to compute the input current fed to the IF neurons:

(10) $$g_{\text{IF}}(S_{k}^{p})=G_{\text{min}}+\frac{G_{\text{max}}-G_{\text{min}}}{1+\text{exp}(-(S_{k}^{p}-x_{0})/\lambda )}.$$

*G*_min_ and *G*_max_ define the interval in which the output firing rates of the IF neurons are taking values. They are set experimentally to achieve spike rates spanning from 5 kHz to 15 kHz. λ sets the selectivity of the sigmoid which is an increasing function of λ (λ has been experimentally tuned to 5.0*e*^−5^ in our experiments). The value of the offset *x*_0_, which is subtracted from the *S*^*p*^_*k*_ is managed by a proportional controller. Its input reference is set such that *x*_0_ approaches the value of *S*^*p*^_*k*_ output by the selected best predictor. This ensures that whatever the current state of the system is, the sigmoid *g*_IF_ is always centered on the current value of interest, giving the best selectivity possible to detect changes in the best predictor. The update period of this controller is set to 0.5 ms. The index of the spiking neuron from the WTA network then corresponds to the best predictor *W*(*t*) satisfying:

(11) $$W(t)=\underset{k\;\in \;\{1,\ldots ,N\}}{\text{argmax}}g_{IF}(S_{k}^{p}(t))\;\;\;.$$

The obtained index *W*(*t*) is used to drive the learning process of the *ESN layer*. Only the ESN selected by the WTA network (ESN with index *W*(*t*)) is trained on the input signal. This adaptive WTA achieves good performance in the selection of the best predictor even if the similarity measurement has a large variance (this happens for instance if the system is exposed to a set of very different stimuli).

This setup of the WTA architecture always generates outputs, even if no input is present. This potential inefficiency can be avoided by adding another output layer, which computes a gating function that depends on the global input activity. Using this mechanism, output neurons driven by the output of the WTA will only fire if in addition the input activity is bigger than a defined threshold. The threshold can be either defined on the average event rate, or the average value of *A*(*t*_*n*_).

### 2.6. Predictability minimization

The third layer implements, in addition to the WTA selection, a predictability minimization algorithm, which ensures that each ESN specializes in predicting different features in the input. It implements a criterion suggested by Barlow (1989) and Schmidhuber (1991) to evaluate the relevance of the prediction of each ESN: an ESN's prediction is considered relevant if it is not redundant given the other ESNs' predictions. This predictability minimization step promotes orthogonality of predictions between the individual ESNs, and encourages a maximally sparse representation of the learned input classes, thereby achieving good coverage of the presented input space. For each ESN *k*, an estimator *Ŵ*_*k*_ of the WTA output is used, which receives only the similarity measures *S*^*p*^_*k*′_ of the other ESNs as input. For a consistent framework of estimators and predictors, we chose to use ESNs (named *PM*_1_, …, *PM*_*N*_ in Figure 2) to implement the *Ŵ*_*k*_ estimator. This also allows taking into account the highly dynamic information contained in the input data recorded with the DVS. Training of the ESNs follows the same principles as described in Section 2.4.

If the estimator *Ŵ*_*k*_ and the WTA output agree, i.e., *Ŵ*_*k*_(*t*_*n*_) = *W*(*t*_*n*_), then this means that the *k*^th^ ESN is not currently learning a new feature, because the same information can also be deduced from the output of the other ESNs. In this case, the corresponding neuron of the WTA is inhibited to prevent this ESN from learning the currently presented input patter. The inhibition also causes the output of the WTA to stop responding to the input, thus promoting another one.

## 3. Results

### 3.1. Experimental setup

**Figure 4:**
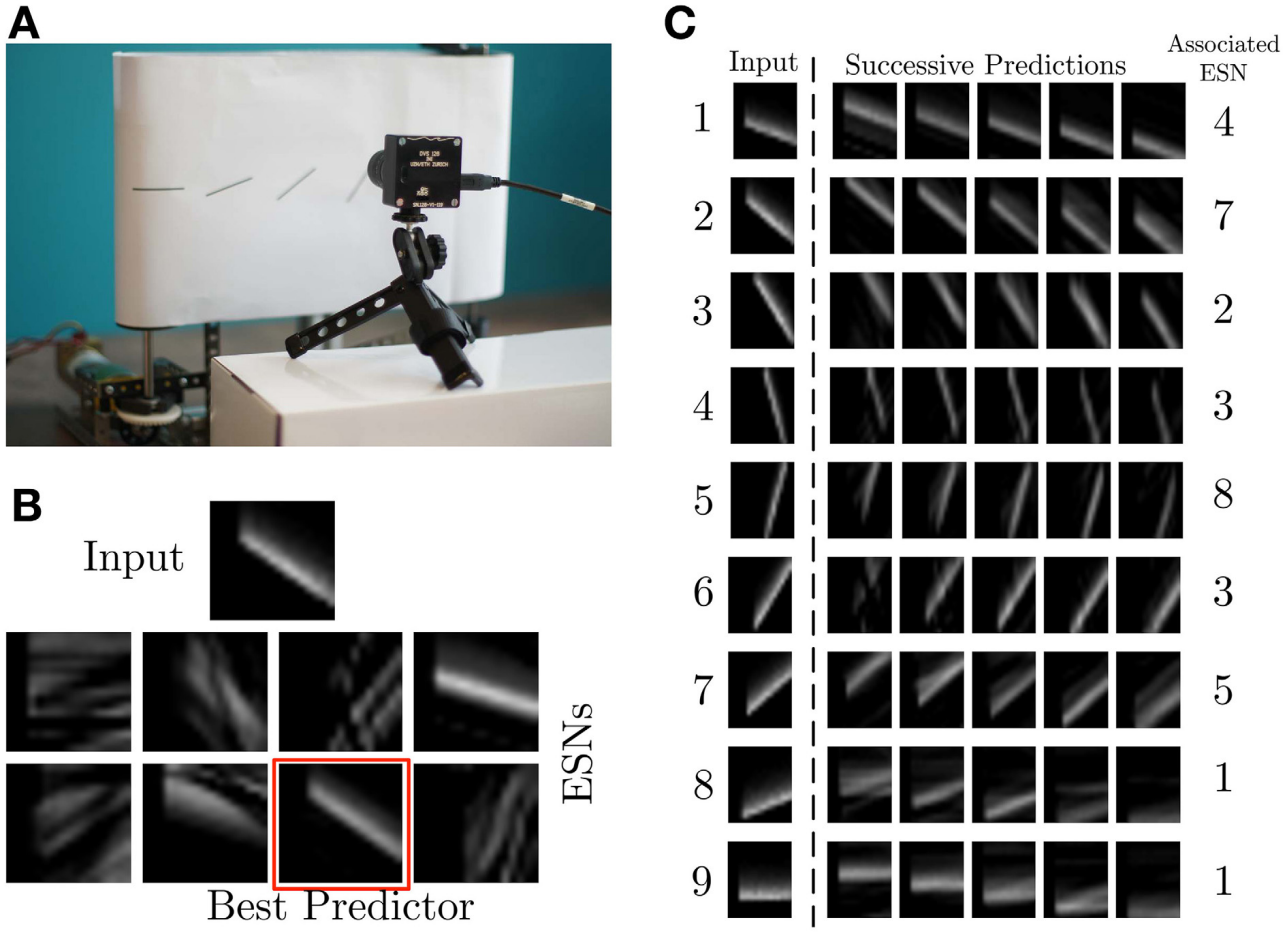
**Experimental recording setup**. **(A)** A DVS records patterns moving on a treadmill. **(B)** Current input pattern (top) and predictions of the different ESNs at a given time. The best predictor is highlighted in red. **(C)** Results of ESN training. The left column shows a snapshot from each of the nine different patterns. The plots to the right show different predictions for different time steps in the future, obtained from the ESN which is specialized in the given pattern. The time difference between the five predicted patterns is 0.01 s.

The experiments presented in this article were performed with the setup shown in Figure 4A. It consists of a DVS retina observing a treadmill, on which moving bars with 9 different orientations move across the field of view of the DVS at constant speed. For the experiments, the recurrent connectivity matrix *W*_r_ for each ESN was initialized randomly, and rescaled to have spectral radius 0.7, which fulfills the Echo State Property (Jaeger, 2002). The other weight matrices were randomly chosen from a uniform distribution in [−0.4; 0.4] for *W*_in_, [−0.02, 0.02] for *W*_back_ and [−0.01, 0.01] for *W*_out_. The pre-processing uses exponential kernels with a time constant of 10 ms.

### 3.2. Single receptive field

The first experiment uses 8 ESNs, each composed of 15 analog neurons, randomly connected in the reservoir. Only one RF, consisting of 17 × 17 cells *C*(*x*_*c*_, *y*_*c*_) spanning 5 × 5 pixel is used as input to each ESN. Figure 4B shows the different predictions of the ESNs in response to an input signal. The WTA succeeds in selecting the best predicting network for the current input. Figure 4C shows for each stimulus the best predictions and the associated ESN. As expected, the results confirm that every network has specialized in the prediction of the temporal evolution of a specific oriented moving pattern. Since natural scenes contain many independent features, which are likely to occur in larger numbers than the number of available ESNs, we tested here the performance of an architecture with only 8 ESNs for 9 different patterns of moving oriented bars. The results indicate that some of the ESNs tend to learn more than one dynamic feature, so that the system can represent all input features as accurately as possible. In order to select the most appropriate number of predictors, additional control mechanisms could be employed. An example of this is the response of ESN1, which is the best predictor both for pattern 8 and 9 (Figure 4C).

**Figure 5:**
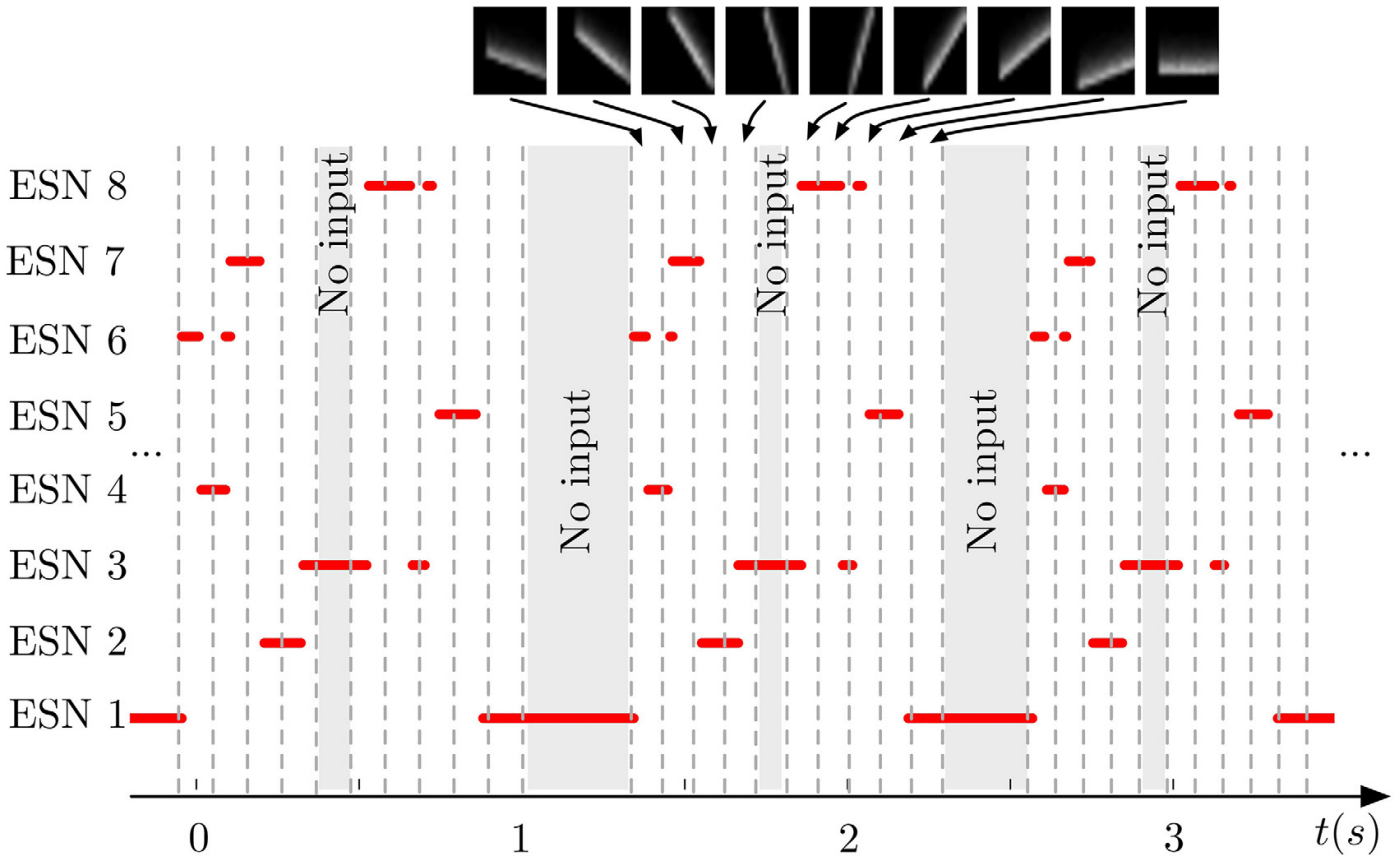
**Output of the WTA network during repeated presentation of a series of nine input patterns**. Red lines indicate when an ESN was selected by the WTA network. Dashed vertical lines mark time points when the input presented to the DVS changed from one pattern to another (the 9 patterns are shown on top of the figure). Shaded areas indicate times when no stimulation was present. Every ESN learns to respond to only a small subset of input patterns (typically exactly one pattern). This response is reproducible over different stimulus presentations.

Figure 5 shows the output of the same system for three successive testing presentations of the stimulus. We can see that each ESN is responding to a specific orientation of the bars. Moreover, the process is repeatable over the three presentations with a difference in the temporal span of the responses. This is due to the increase of the translation speed of the bars during the recording to show that the networks effectively respond to the bar's orientations independently of their speed.

**Figure 6:**
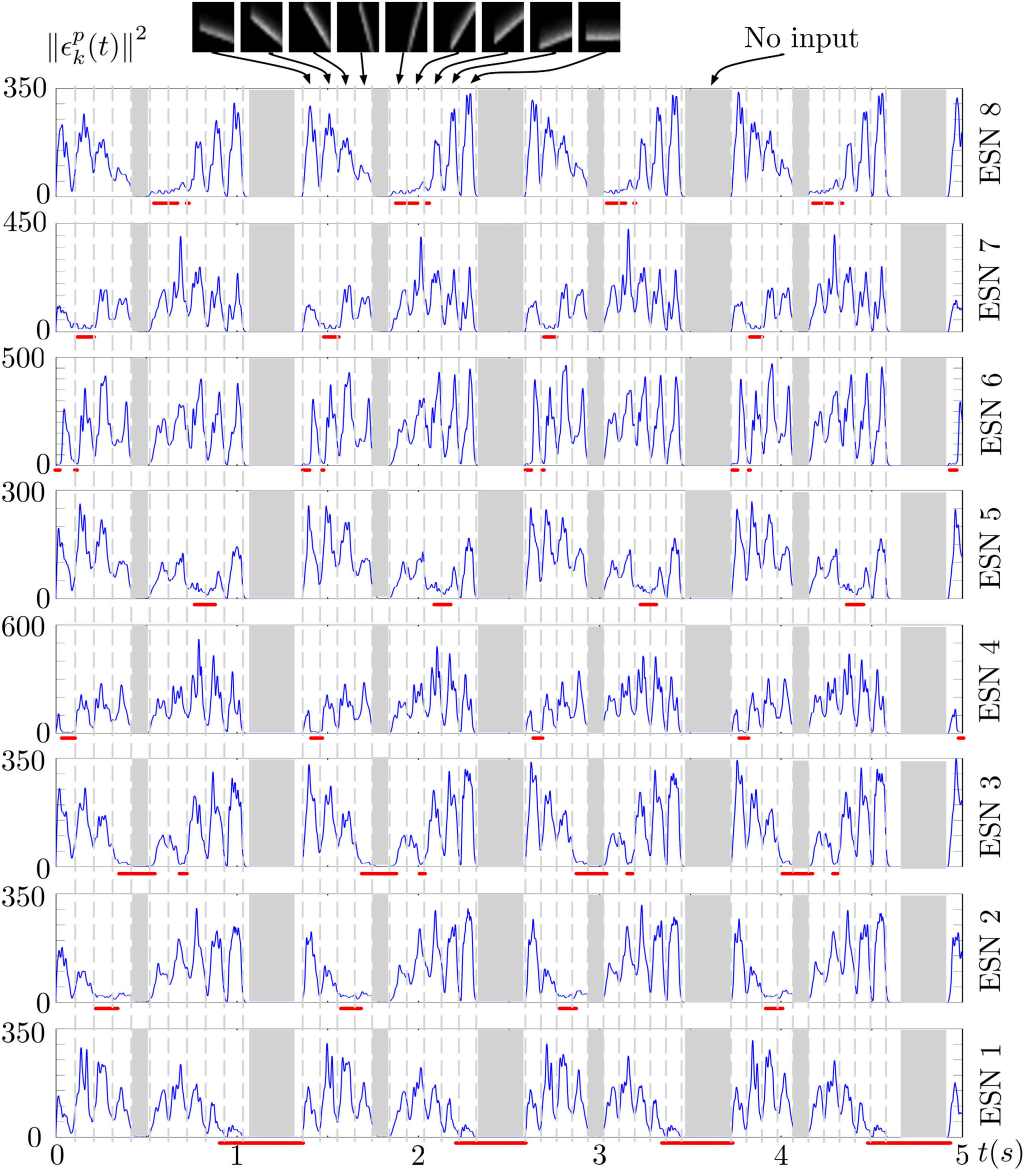
**Prediction error of the 8 ESNs during several presentations of the input stimulus**. Red lines below each plot shows the output of the WTA neuron corresponding to each ESN, thus indicating times when each ESN was selected as the best predictor. We can observe that ESNs are correctly selected when their prediction error is minimal amongst all the networks.

Figure 6 shows the prediction error of each ESN during several presentations of the stimulus. The output of the WTA network is shown below each curve, indicating when a particular ESN is selected as the best predictor. An ESN is correctly selected whenever its prediction error is the lowest. Periods in which all prediction errors are close to zero correspond to periods without input (shown as gray regions in the figure). This is a result of the approximate linearity of the ESNs and their low spectral radius: when only weak input is fed into the network, the ESNs readout output also approaches zero, which results in a low prediction error for times when no stimulus is presented (the only input to the networks then is background noise from the DVS pixels).

### 3.3. Multiple receptive fields

**Figure 7:**
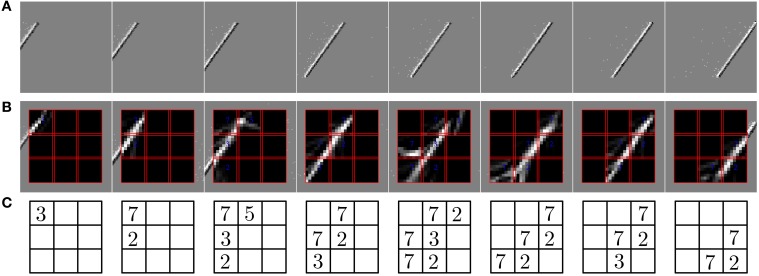
**Predictions of the ESNs when the camera's field of view is divided into multiple receptive fields (RFs)**. **(A)** Output of the DVS for a bar moving across the field of view. **(B)** Predictions of ESNs processing different RFs (indicated by red boxes). **(C)** Index of the best predicting ESN in every RF at the timepoints of the snapshots. Indices are only shown if the input activity in the RF exceeds a given threshold.

**Figure 8:**
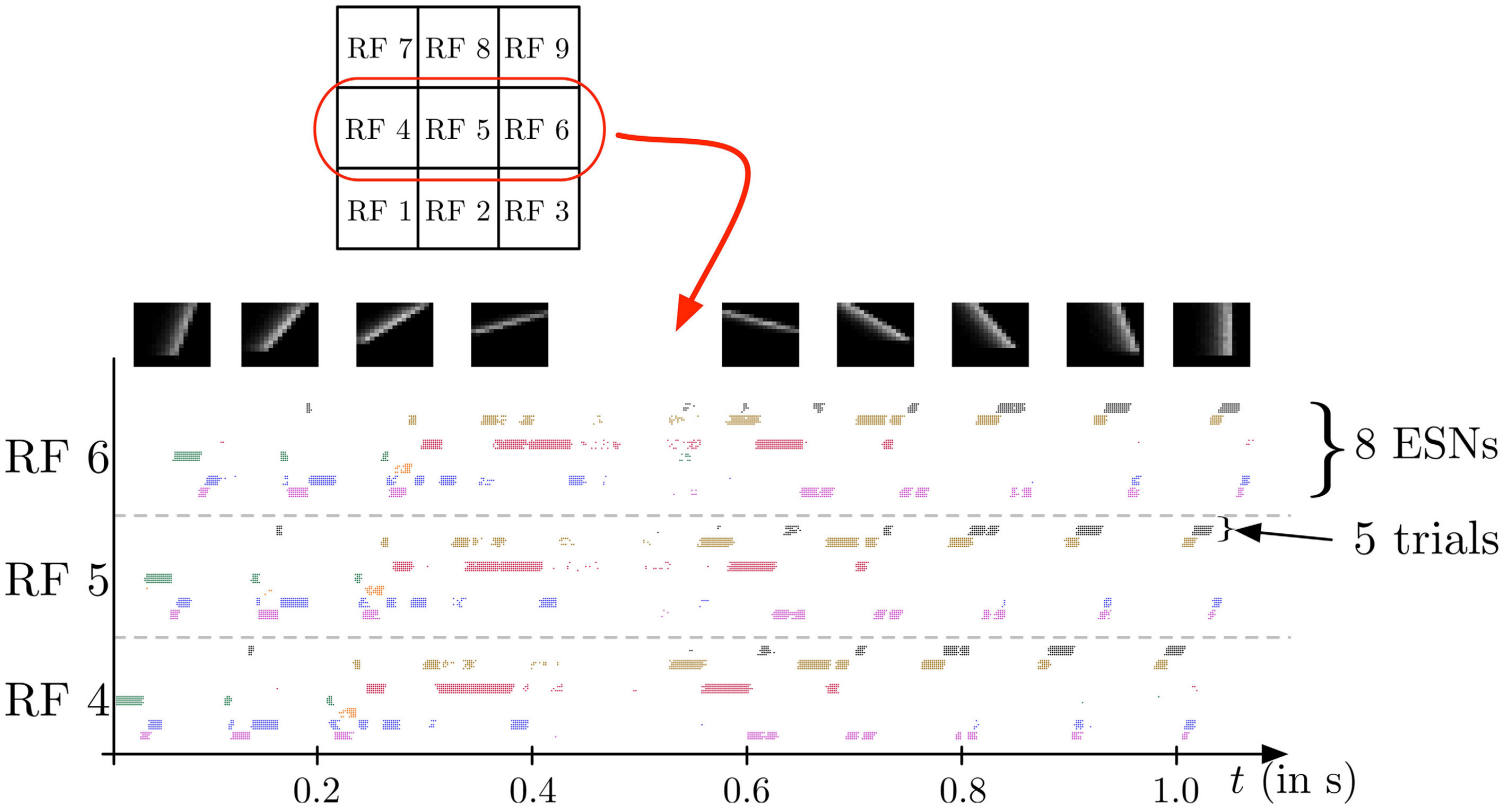
**Spike output for the WTA neurons corresponding to the eight ESNs for each of the 3 central RFs (see Figure 7) during the presentation of a series of 9 moving bars with different orientations (see snapshots on top)**. Dots indicate the times of output spikes for 5 repeated stimulus presentations, which are drawn on different coordinates along the y-axis, but grouped by WTA neuron. Each ESN, depending on its index in the RF, is associated with a color used to represent the dots corresponding to its output. The results show a highly reproducible response of the feature detectors for different trials, and also similar time delays for different stimulus presentations. Because the input stimulus moves horizontally, the outputs of the WTA circuits are similar, with a little time delay. Note that only one ESN can be active at any given time in each RF. Apparent simultaneous spikes from multiple WTA neurons are due to the scaling of time in the horizontal axis of the figure.

In the second experiment, the field of view of the DVS is split into 3× 3 smaller RFs of identical size (9 × 9 cells of 3 × 3 pixels), as shown in Figure 7. This shows the full intended behavior of the system as a local spatiotemporal feature detector, in which different features can be assigned to small receptive fields covering the entire field of view of the sensor (instead of being covered by only one big one RF like in Figure 4). For each RF 8 ESNs are used as feature detectors. In the learning phase, they are trained only with the input to the central RF. Subsequently, their weights are copied and the ESNs are used independently for all 9 RFs. Thus, all RFs have ESNs with identical weights (and so detects the same features), but receive different inputs and therefore evolve independently. Figure 7A shows different snapshots of the DVS recording for an oriented bar moving across the field of view. The output of the predictors for each RF is shown in Figure 7B, while Figure 7C indicates for each RF the index of the ESN selected. The figure also shows that ESN predictors are only selected when there is substantial input activity in the RF. As in the previous experiment, dynamic feature selection is reproducible and exhibits precise timing, as shown in Figure 8. Here, only the 3 RFs on the middle line of the input space are shown. Because the input stimulus moves horizontally, the outputs of the WTA circuits are similar, with a little time delay. Using multiple smaller RFs instead of one is also a potential solution to represent more features with a finite set of ESN. The feature descriptor is then a combination of the outputs of all available ESNs, which need to be processed by another layer. This is however, beyond the scope of the present paper.

**Figure 9:**
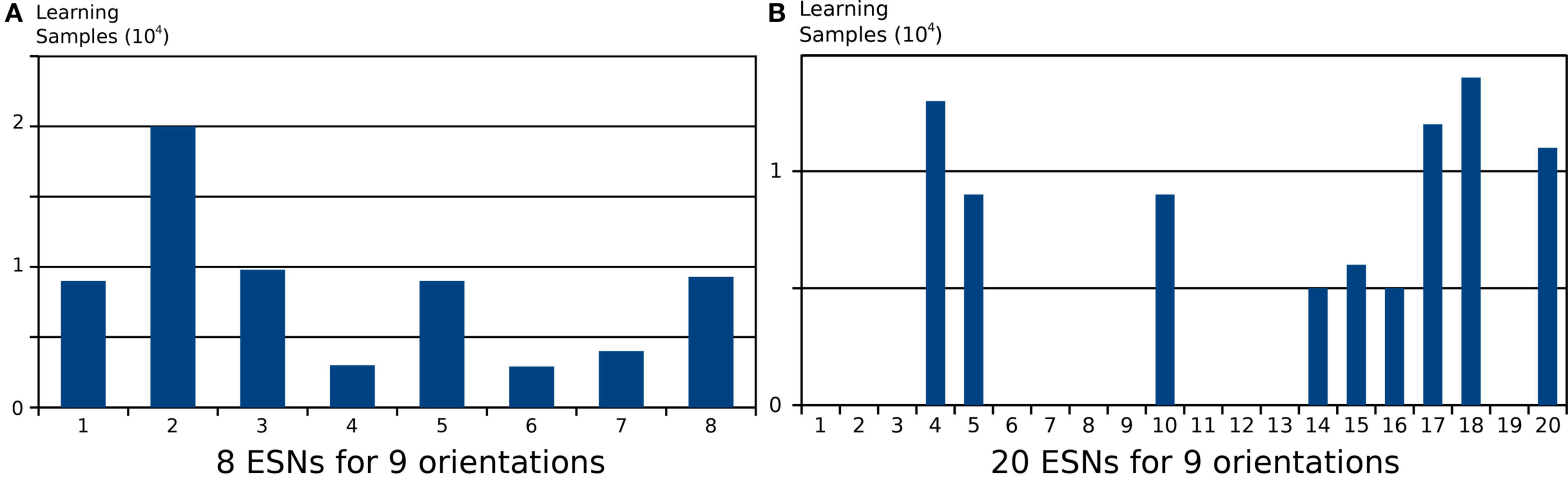
**Number of learning samples per reservoir for two different architectures applied to the same input**. Every step where training of the ESN readout was activated is counted as a learning sample. **(A)** Learning samples for 8 ESNs trained on 9 different input patterns. **(B)** Learning samples for 20 ESNs trained on the same 9 input patterns. The results show that when the pool of ESNs is bigger that the number of features present in the input, only a necessary subset of ESNs from the pool is used to learn these features. The remaining ESNs are not trained, and can be used to learn new features from future inputs.

Choosing the right number of ESNs for the feature detection architecture is not always straightforward, and depends on the number of distinct features present in a scene. In Figure 5 it was shown that when the number of ESNs is smaller than the number of features, an ESN can learn multiple features instead of one. Figure 9A shows the number of steps in which each ESN is trained if 8 ESNs are trained on 9 different input patterns. It is shown that all networks are trained for a similar number of epochs. When instead the number of ESNs exceeds the number of features, we find that only the minimum necessary number of predictors is selected, and the remaining ESNs are still available to learn new features, should there be distinct future visual inputs. Figure 9B a clear specialization of ESNs, if 20 networks are used to encode the same 9 features that were used in Figure 9A. Only 9 out the 20 ESNs show increased activation during the stimulation presentations.

### 3.4. Complex input stimulus

**Figure 10:**
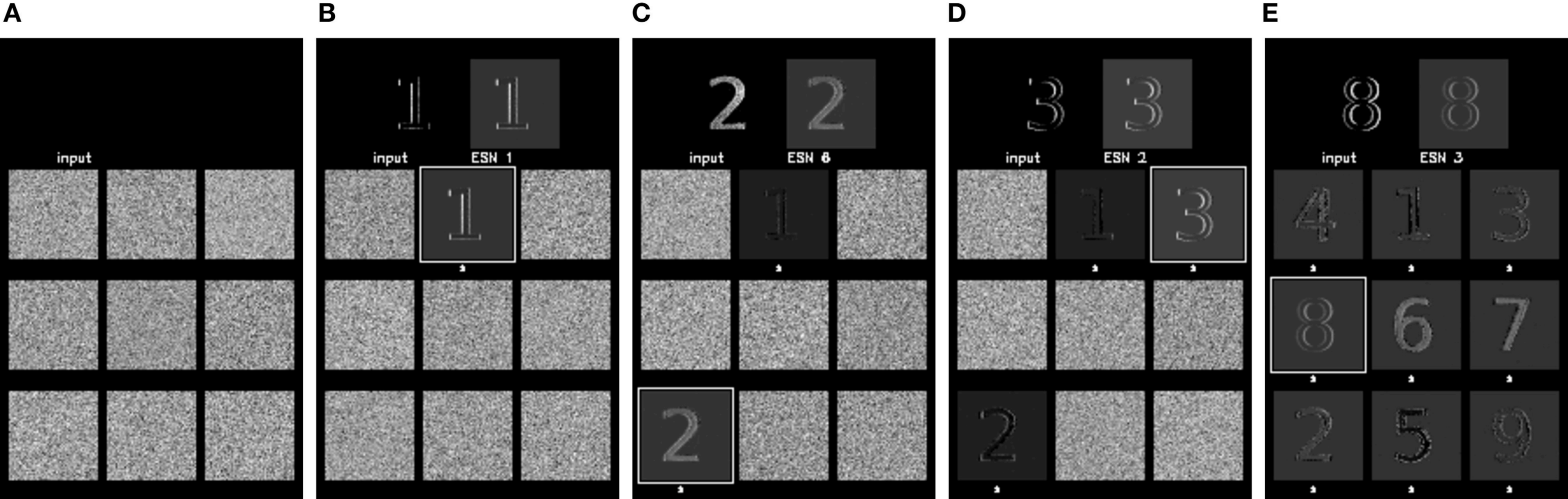
**Learning of more complex feature detectors, showing the output of the system when presented with stimuli composed of digits from 1 to 9**. Each snapshot on the top left shows the analog input to the ESN, obtained by filtering the DVS events. The top right shows the prediction of the best ESN, as selected by the WTA circuit. Below, the current predictions of all nine ESNs used in the experiment are shown. A white square around the prediction indicates the ESN that was selected by the WTA. The snapshots show the progression of learning, starting with an untrained network in **(A)**, which only produces random predictions. **(B–D)** show the output of the same networks after the presentation of patterns “1,” “2,” and “3,” (respectively). A white mark underneath an ESN prediction indicates that this ESN has learned a feature. Finally **(E)** shows its output after the end of the learning process where all networks have learnt an input stimulus.

In the last experiment, the ability of the architecture to represent more complex features was tested. Instead of using oriented bars, we now present digits (from 1 to 9) to the camera, with a single receptive field covering the whole stimulus. Nine ESNs were used in the system, which matches the number of distinct patterns. To make them visible for DVS recordings, the nine digits were animated, by hand, with a random jittering movement around a central spatial position. This was intended to simulate what would be seen by the retina when the eye follows microsaccadic movements. Because the jitter is random, the input stimulus mainly contains spatial information. This experiment allows us to test the robustness of the proposed method to several spatiotemporal patterns, including the degenerate case where only one spatial information is relevant for the feature. Some snapshots of the system's output are shown in Figure 10.

**Figure 11:**
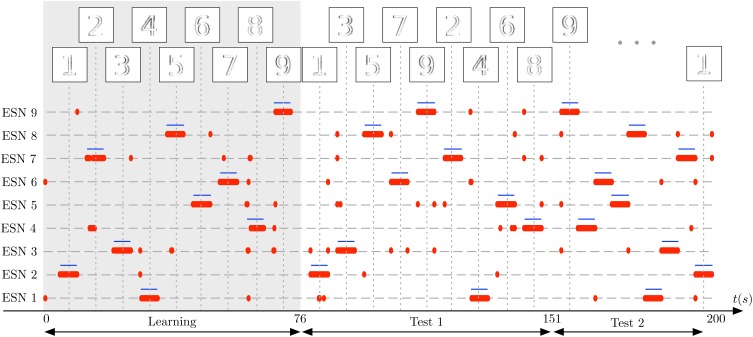
**Learning complex features from DVS inputs**. The DVS records digits from 1 to 9, each animated with random jitter simulating the effect of microsaccades in the biological eye. Blue horizontal lines show the ground truth, indicating when each number was present in the input, and thus when a specific ESN should fire. The corresponding pattern presented to the camera at that time is represented on top of the figure. Red dots show time points where each ESN is selected by the WTA network. The gray shaded area marks the learning phase, in which every digit from 1 to 9 was presented once to the system. Subsequently, two series of tests are shown: First, in the period marked as *Test 1*, the 9 digits are presented in random order, but with short pauses between stimulus presentations. Then, in the period marked as *Test 2*, the digits are presented again in a different random order, but without pauses in between. The results show that also for complex features like digits, every ESN can learn to specialize and represent a distinct feature.

In the first stage of the experiment, the system is presented with visual stimuli of the digits 1–9, in this order. The images at the top of the plot shows the input to the receptive field at the time of the vertical dotted lines. Each number is presented for 5 s, followed by a pause of 3 s, in which no input is presented. In Figure 11 the learning phase is marked by a gray shaded background. Next, two test sequences are presented to the DVS: The *Test 1* sequence is composed of the random sequence “1 3 5 7 9 2 4 6 8,” using the same presentation and pause times as in the learning phase. The *Test 2* sequence is composed of another random sequence “9 8 7 6 5 4 3 2 1,” this time without pauses between digit presentations (which still last for 5 s). These sequences are represented as the ground truth for the experiment by blue horizontal lines in Figure 11. For clarity, we re-ordered the ESNs such that the ESN index corresponds to the digit it represents. Successful learning means that the blue lines should align as much as possible with the red dots, representing the output of the WTA network. Occasional deviations are due to noise.

Figure 11 shows that each ESN manages to learn complex features, and reliably recognizes them when the respective feature is presented again. This was achieved with raw, noisy DVS inputs, and fully random jitter of the digits during presentation. The experiment shows that complex features can be extracted and recognized also in the absence of characteristic spatiotemporal structure in input patterns.

## 4. Discussion

This article presents a new architecture for extracting spatiotemporal visual features from the signal of an asynchronous event-based silicon retina. The spatiotemporal signal feeds into the system through a layer of ESN, which compute predictions of future inputs. An unsupervised learning process leads to specialization of ESNs to different features via WTA competition, which selects only the best predictors of the present input pattern for training. Whenever an already learned pattern is presented again, the system can efficiently and reliably detect it. Experimental results confirm the suitability of the feature extraction method for a variety of input patterns. The spatiotemporal feature extraction leads to robust and reproducible detection, which is a key requirement for its use in higher-level visual recognition and classification. A central characteristic of the presented technique, in contrast to conventional computer vision methods, is that it does not depend on the concept of representing visual inputs as whole image frames. Instead, the method works efficiently on event-based sparse and asynchronous input streams, which maintain the temporal dynamics of the scene due to the highly precise asynchronous time sampling ability of the silicon retina. Thus, also the extracted spatiotemporal features contain richer dynamic information, in addition to recognizing spatial characteristics.

Central to the definition of spatiotemporal features in our architecture is the presence of multiple models for prediction, which compete already during learning, such that specialization can occur. Similar concepts are used by various well-known machine learning frameworks, most notably the mixture-of-experts architecture (Jacobs et al., 1991; Jordan and Jacobs, 1994; Yuksel et al., 2012), in which a gating function creates a soft division of the input space for multiple local “expert” models. The output of the whole network is then a combination of the expert predictions, weighted according to their responsibility for the present input. These architectures have been extended in brain-inspired architectures for reinforcement learning and control (Haruno et al., 2001; Doya et al., 2002; Uchibe and Doya, 2004), where multiple forward models and controllers are learned simultaneously, and the prediction performance of the forward model determines the selection of the most appropriate local controller. Mixture-of-experts architectures are closely related to learning mixture models with the EM algorithm (Dempster et al., 1977; Jordan and Jacobs, 1994), where the E-step computes a soft assignment of data points to models. Nessler et al. (2009) and Nessler et al. (2013) have proven that this can be implemented in spiking neural networks, using a soft WTA circuit to compute the E-step, and an STDP learning rule to implement the M-step. Compared to these related architectures, our new model advances in three important aspects: Firstly, whereas EM and mixture-of-experts address static input distributions, we here extend this to multiple feature predictors for spatiotemporal sequences. Secondly, our architecture allows online learning of independent features, which contrasts with batch methods like PCA or ICA that operate on the full dataset after its collection. Thirdly, our neural network architecture is specifically designed to work with spiking inputs and for implementation with spiking neurons, thus maintaining the precise dynamics of event-based vision sensors. Other spiking neural network architectures for processing DVS inputs such as spiking ConvNets (Farabet et al., 2012; Camuñas-Mesa et al., 2014), and spiking Deep-belief networks (O'Connor et al., 2013) do not explicitly model the dynamics of the features extracted within the networks, but instead rely on different conversion mechanisms from analog to spiking neural networks, without taking sensor dynamics into account. The features they extract are thus characterizing a current snapshot of the input, and do not take its future trajectory into account like the ESN predictors of the presented model, but nevertheless are very useful for fast recognition. This is also true for approaches that directly classify spatiotemporal spike patterns, see e.g., (Sheik et al., 2013; Tapson et al., 2013). Spiking network models that represent spatiotemporal dynamics by emulating Hidden Markov Models have recently been introduced (Corneil et al., 2014; Kappel et al., 2014). Compared to our approach, these networks do not directly learn dynamic input features, but rather identify hidden states to determine the position within longer sequences.

The combination of visual sensing with bio-inspired artificial retinas and event-based visual feature extraction, as presented in this article, opens new perspectives for apprehending the mechanisms of visual information encoding in the brain. It is clear that the traditional views of visually selective neurons as static image filters for receptive fields, e.g., as Gabor-like orientation filters, which are central to many classical vision models like HMAX or Neocognitron (Fukushima, 1980; Serre et al., 2002), fails to explain how these neurons deal with the highly dynamic and sparse spike inputs from biological retinas. In the presented approach, features are naturally learned and adapted to the task. In Figure 9 it was shown that if the number of available ESNs exceeds the number of features necessary to describe a scene, only the minimum necessary number of networks are trained. This has the desirable effect that whenever a new scene with new features is encountered, the previously unused ESNs can be trained to predict novel stimulus features. This behavior has several benefits: firstly, the number of ESNs does not have to be precisely tuned, but can be set to the highest acceptable number, and only the minimum number of networks is actually recruited and trained as feature detectors by the system. Alternatively, one could employ a different strategy in which new networks are recruited to the pool, whenever all current ESNs have specialized on features. Secondly, training of feature detectors works completely unsupervised, so no higher-level controller is needed to identify what the elementary features for a scene should be. Although the precesence of a supervisor is not necessary, having such information available would still be beneficial. For instance, another processing layer could use the outputs of the WTA to control the survival of each network. If such processing layer determines that a particular network does not provide enough interesting information, the supervisor could decide to reset and release the associated ESN, so that it can detect more relevant features.

The presented method has great potential for use in event-based vision applications, such as fluid and high-speed recognition of objects and sequences, e.g., in object and gesture recognition (O'Connor et al., 2013; Lee et al., 2014), or for high-speed robotics (Conradt et al., 2009; Mueggler et al., 2014).

The presented architecture is almost entirely based on computation with spikes. Inputs come in the form of AER events from DVS silicon retinas, providing an event-based representation of the visual scene. The WTA circuit for choosing between feature extractors is also working with spikes, and produces spike outputs, which indicate the identity of the detected feature. The only component of the system which does not entirely use spikes is the layer of ESNs that predict the visual input, but this restriction could be lifted by replacing ESNs with their spiking counterparts, called Liquid State Machines (LSMs) (Maass et al., 2002), which are computationally at least equivalent to ESNs (Maass and Markram, 2004; Büsing et al., 2010). The reasons why we have chosen to use ESNs for this proof-of-principle study are the added difficulty of tuning LSMs, due to the larger number of free parameters for spiking neuron models, delays, or time constants, in addition to the higher computational complexity involved in the simulation of spiking neural networks on conventional machines, which makes it hard to simulate multiple LSMs in real-time. Overall, we expect the improvement due to using fully spike-based feature detectors and predictors to be rather minor, since the ESNs can be efficiently simulated at time steps of 1 ms, which is also the time interval at which the silicon retina is sending events through the USB bus. However, a fully spike-based architecture does have great advantages in terms of efficiency and real-time executing if it can be implemented entirely on configurable neuromorphic platforms with online learning capabilities (Indiveri et al., 2006; Galluppi et al., 2014; Rahimi Azghadi et al., 2014), which is the topic of ongoing research.

### Conflict of interest statement

The Reviewer Federico Corradi declares that, despite being affiliated to the same institution as author Michael Pfeiffer, the review process was handled objectively and no conflict of interest exists. The authors declare that the research was conducted in the absence of any commercial or financial relationships that could be construed as a potential conflict of interest.

## References

[B1] Barlow H. B. (1989). Unsupervised learning. Neural Comput. 1, 295–311. 10.1162/neco.1989.1.3.29518282822

[B2] Bay H. Ess A. Tuytelaars T. Van Gool L. (2008). Speeded-up robust features (SURF). Comput. Vis. Image Underst. 110, 346–359 10.1016/j.cviu.2007.09.014

[B3] Berry M. Warland D. K. Meister M. (1997). The structure and precision of retinal spike trains. Proc. Natl. Acad. Sci. U.S.A. 94, 5411–5416. 10.1073/pnas.94.10.5411 9144251 PMC24692

[B4] Büsing L. Schrauwen B. Legenstein R. (2010). Connectivity, dynamics, and memory in reservoir computing with binary and analog neurons. Neural Comput. 22, 1272–1311. 10.1162/neco.2009.01-09-947 20028227

[B5] Camuñas-Mesa L. A. Serrano-Gotarredona T. Ieng S. H. Benosman R. B. Linares-Barranco B. (2014). On the use of orientation filters for 3d reconstruction in event-driven stereo vision. Front. Neurosci. 8:48. 10.3389/fnins.2014.00048 24744694 PMC3978326

[B6] Conradt J. Cook M. Berner R. Lichtsteiner P. Douglas R. J. Delbruck T. (2009). A pencil balancing robot using a pair of aer dynamic vision sensors, in IEEE International Symposium on Circuits and Systems (ISCAS) (Taipei), 781–784.

[B7] Corneil D. S. Neftci E. Indiveri G. Pfeiffer M. (2014). Learning, inference, and replay of hidden state sequences in recurrent spiking neural networks, in Computational and Systems Neuroscience (COSYNE) (Salt Lake City, UT), 1–2.

[B8] Coultrip R. Granger R. Lynch G. (1992). A cortical model of winner-take-all competition via lateral inhibition. Neural Netw. 5, 47–54 10.1016/S0893-6080(05)80006-1

[B9] Delbruck T. Linares-Barranco B. Culurciello E. Posch C. (2010). Activity-driven, event-based vision sensors, in Proceedings of 2010 IEEE International Symposium on Circuits and Systems (ISCAS) (Paris), 2426–2429 10.1109/ISCAS.2010.5537149

[B10] Dempster A. P. Laird N. M. Rubin D. B. (1977). Maximum likelihood from incomplete data via the EM algorithm. J. R. Stat. Soc. B 39, 1–38.

[B11] Douglas R. J. Mahowald M. A. Martin K. A. C. (1994). Hybrid analog-digital architectures for neuromorphic systems, in Proceedings of 1994 IEEE World Congress on Computational Intelligence (Orlando, FL), 1848–1853.

[B12] Doya K. Samejima K. Katagiri K.-I. Kawato M. (2002). Multiple model-based reinforcement learning. Neural Comput. 14, 1347–1369. 10.1162/089976602753712972 12020450

[B13] Farabet C. Paz R. Pérez-Carrasco J. Zamarreño-Ramos C. Linares-Barranco A. LeCun Y. (2012). Comparison between frame-constrained fix-pixel-value and frame-free spiking-dynamic-pixel convnets for visual processing. Front. Neurosci. 6:32 10.3389/fnins.2012.00032 PMC3324817 22518097

[B14] Farhang-Boroujeny B. (2013). Adaptive Filters: Theory and Applications. Hoboken, NJ: John Wiley & Sons 10.1002/9781118591352

[B15] Fukushima K. (1980). Neocognitron: a self-organizing neural network model for a mechanism of pattern recognition unaffected by shift in position. Biol. Cybern. 36, 193–202. 7370364 10.1007/BF00344251

[B16] Gabor D. (1946). Theory of communication. J. IEEE 93, 429–459.

[B17] Galluppi F. Lagorce X. Stromatias E. Pfeiffer M. Plana L. A. Furber S. B.. (2014). A framework for plasticity implementation on the SpiNNaker neural architecture. Front. Neurosci. 8:429. 10.3389/fnins.2014.00429 25653580 PMC4299433

[B18] Gollisch T. Meister M. (2008). Rapid neural coding in the retina with relative spike latencies. Science 319, 1108–1111. 10.1126/science.1149639 18292344

[B19] Haruno M. Wolpert D. Kawato M. (2001). MOSAIC model for sensorimotor learning and control. Neural Comput. 13, 2201–2220. 10.1162/089976601750541778 11570996

[B20] Huang L. Shimizu A. Kobatake H. (2004). Classification-based face detection using Gabor filter features, in Proceedings of Sixth IEEE International Conference on Automatic Face and Gesture Recognition (Seoul), 397–402.

[B21] Hubel D. H. Wiesel T. N. (1962). Receptive fields, binocular interaction and functional architecture in the cat's visual cortex. J. Physiol. 160, 106–154. 10.1113/jphysiol.1962.sp006837 14449617 PMC1359523

[B22] Ilonen J. Kamarainen J.-K. Kalviainen H. (2007). Fast extraction of multi-resolution gabor features, in 14th International Conference on Image Analysis and Processing (ICIAP) (Modena), 481–486 10.1109/ICIAP.2007.4362824

[B23] Indiveri G. Chicca E. Douglas R. (2006). A VLSI array of low-power spiking neurons and bistable synapses with spike-timing dependent plasticity. IEEE Trans. Neural Netw. 17, 211–221. 10.1109/TNN.2005.860850 16526488

[B24] Jacobs R. A. Jordan M. I. Nowlan S. J. Hinton G. E. (1991). Adaptive mixtures of local experts. Neural Comput. 3, 79–87 10.1162/neco.1991.3.1.79 31141872

[B25] Jaeger H. (2002). Tutorial on Training Recurrent Neural Networks, Covering bppt, rtrl, ekf and the “Echo State Netwrok” Approach. Technical report, German National Research Center for Information Technology.

[B26] Jaeger H. Haas H. (2004). Harnessing nonlinearity: predicting chaotic systems and saving energy in wireless communication. Science 304, 78–80. 10.1126/science.1091277 15064413

[B27] Jordan M. I. Jacobs R. A. (1994). Hierarchical mixtures of experts and the EM algorithm. Neural Comput. 6, 181–214. 10.1162/neco.1994.6.2.18118263525

[B28] Kappel D. Nessler B. Maass W. (2014). STDP installs in winner-take-all circuits an online approximation to hidden markov model learning. PLoS Comput. Biol. 10:e1003511. 10.1371/journal.pcbi.1003511 24675787 PMC3967926

[B29] Lee J. H. Delbruck T. Pfeiffer M. Park P. K. Shin C.-W. Ryu H.. (2014). Real-time gesture interface based on event-driven processing from stereo silicon retinas. IEEE Trans. Neural Netw. Learn. Syst. 25, 2250–2263. 10.1109/TNNLS.2014.2308551 25420246

[B30] Lichtsteiner P. Posch C. Delbruck T. (2008). A 128X128 120dB 15us latency asynchronous temporal contrast vision sensor. IEEE J. Solid State Circ. 43, 566–576 10.1109/JSSC.2007.914337

[B31] Lin C. Huang C. (2005). A complex texture classification algorithm based on gabor-type filtering cellular neural networks and self-organized fuzzy inference neural networks, in IEEE International Symposium on Circuits and Systems (ISCAS) (Kobe), 3942–3945.

[B32] Liu S.-C. Oster M. (2006). Feature competition in a spike-based winner-take-all VLSI network, in Proceedings of 2006 IEEE International Symposium on Circuits and Systems (ISCAS) (Kos), 3634–3637.

[B33] Lowe D. G. (1999). Object recognition from local scale-invariant features, in The Proceedings of the Seventh IEEE International Conference on Computer Vision (ICCV) (Kerkyra), 1150–1157.

[B34] Lowe D. G. (2004). Distinctive image features from scale-invariant keypoints. Int. J. Comput. Vis. 60, 91–110. 10.1023/B:VISI.0000029664.99615.9417946463

[B35] Maass W. Markram H. (2004). On the computational power of circuits of spiking neurons. J. Comput. syst. Sci. 69, 593–616 10.1016/j.jcss.2004.04.001

[B36] Maass W. Natschläger T. Markram H. (2002). Real-time computing without stable states: a new framework for neural computation based on perturbations. Neural Comput. 14, 2531–2560. 10.1162/089976602760407955 12433288

[B37] Mead C. Mahowald M. (1988). A silicon model of early visual processing. Neural Netw. 1, 91–97 10.1016/0893-6080(88)90024-X

[B38] Mueggler E. Huber B. Scaramuzza D. (2014). Event-based, 6-DOF pose tracking for high-speed maneuvers, in IEEE/RSJ International Conference on Intelligent Robots and Systems (IROS), 2761–2768.

[B39] Mutch J. Knoblich U. Poggio T. (2010). CNS: a GPU-Based Framework for Simulating Cortically-Organized Networks. Cambridge, MA: CBCL 286, MIT Computer Science and Artificial Intelligence Laboratory, Massachusetts Institute of Technology.

[B40] Nessler B. Pfeiffer M. Buesing L. Maass W. (2013). Bayesian computation emerges in generic cortical microcircuits through spike-timing-dependent plasticity. PLoS Comput. Biol. 9:e1003037. 10.1371/journal.pcbi.1003037 23633941 PMC3636028

[B41] Nessler B. Pfeiffer M. Maass W. (2009). STDP enables spiking neurons to detect hidden causes of their inputs, in Proceedings of Neural Information Processing Systems (NIPS) (Vancouver, BC), 1357–1365.

[B42] O'Connor P. Neil D. Liu S.-C. Delbruck T. Pfeiffer M. (2013). Real-time classification and sensor fusion with a spiking deep belief network. Front. Neurosci. 7:178. 10.3389/fnins.2013.00178. 24115919 PMC3792559

[B43] Olshausen B. A. Field D. J. (1997). Sparse coding with an overcomplete basis set: a strategy employed by V1? Vis. Res. 37, 3311–3326. 10.1016/S0042-6989(97)00169-7 9425546

[B44] Oster M. Douglas R. J. Liu S.-C. (2009). Computation with spikes in a winner-take-all network. Neural Comput. 21, 2437–2465. 10.1162/neco.2009.07-08-829 19548795

[B45] Posch C. Matolin D. Wohlgenannt R. (2011). A QVGA 143 dB Dynamic Range Frame-Free PWM Image Sensor With Lossless Pixel-Level Video Compression and Time-Domain CDS. IEEE J. Solid State Circ. 46, 259–275 10.1109/JSSC.2010.2085952

[B46] Posch C. Serrano-Gotarredona T. Linares-Barranco B. Delbruck T. (2014). Retinomorphic event-based vision sensors: bioinspired cameras with spiking output. Proc. IEEE 102, 1470–1484 10.1109/JPROC.2014.2346153

[B47] Rahimi Azghadi M. Iannella N. Al-Sarawi S. F. Indiveri G. Abbott D. (2014). Spike-based synaptic plasticity in silicon: design, implementation, application, and challenges. Proc. IEEE 102, 717–737 10.1109/JPROC.2014.2314454

[B48] Riesenhuber M. Poggio T. (1999). Hierarchical models of object recognition in cortex. Nat. Neurosci. 11, 1019–1025. 10526343 10.1038/14819

[B49] Roska B. Werblin F. (2003). Rapid global shifts in natural scenes block spiking in specific ganglion cell types. Nat. Neurosci. 6, 600–608. 10.1038/nn1061 12740583

[B50] Schmidhuber J. (1991). Learning factorial codes by predictability minimization. Neural Comput. 4, 863–879 10.1162/neco.1992.4.6.863

[B51] Schrauwen B. Verstraeten D. Campenhout J. V. (2007). An overview of reservoir computing: theory, applications and implementations, in Proceedings of the 15th European Sympsosium on Artificial Neural Networks (Bruges), 471–482.

[B52] Serre T. Bileschi S. Wolf L. Riesenhuber M. Poggio T. (2006). Robust object recognition with cortex-like mechanisms. IEEE Trans. Pattern Anal. Mach. Intell. 29, 2007. 10.1109/TPAMI.2007.56 17224612

[B53] Serre T. Riesenhuber M. Louie J. Poggio T. (2002). On the role of object-specific features for real world object recognition in biological vision, in Proceedings of Biologically Motivated Computer Vision (Tübingen), 387–397.

[B54] Sheik S. Pfeiffer M. Stefanini F. Indiveri G. (2013). Spatio-temporal spike pattern classification in neuromorphic systems, in Biomimetic and Biohybrid Systems, eds Lepora N. F. Mura A. Krapp H. G. Verschure P. F. M. J. Prescott T. J. (London: Springer), 262–273.

[B55] Tapson J. C. Cohen G. K. Afshar S. Stiefel K. M. Buskila Y. Wang R. M.. (2013). Synthesis of neural networks for spatio-temporal spike pattern recognition and processing. Front. Neurosci. 7:153. 10.3389/fnins.2013.00153. 24009550 PMC3757528

[B56] Uchibe E. Doya K. (2004). Competitive-cooperative-concurrent reinforcement learning with importance sampling, in Proceedings of International Conference on Simulation of Adaptive Behavior: From Animals and Animats (Los Angeles, CA), 287–296.

[B57] Uzzell U. J. Chichilnisky E. J. (2004). Precision of spike trains in primate retinal ganglion cells. J. Neurophysiol. 92, 780–789. 10.1152/jn.01171.2003 15277596

[B58] Wallis G. Bülthoff H. (1999). Learning to recognize objects. Trends Cogn. Sci. 3, 22–31. 10.1016/S1364-6613(98)01261-3 10234223

[B59] Wallis G. Rolls E. (1997). A model of invariant object recognition in the visual system. Prog. Neurobiol. 51, 167–194. 10.1016/S0301-0082(96)00054-8 9247963

[B60] Yuksel S. E. Wilson J. N. Gader P. D. (2012). Twenty years of mixture of experts. IEEE Trans. Neural Netw. Learn. Syst. 23, 1177–1193. 10.1109/TNNLS.2012.2200299 24807516

